# Acyl-CoA thioesterase 7 is oncogenic in breast cancer by promoting oxidative phosphorylation via PGC1α

**DOI:** 10.1016/j.gendis.2023.101149

**Published:** 2023-10-20

**Authors:** Xiangyu Sun, Qiang Zhang, Mozhi Wang, Litong Yao, Xiang Li, Hongyi Cao, Yingying Xu

**Affiliations:** a Department of Breast Surgery, Liaoning Cancer Hospital & Institute, Shenyang, Liaoning 110000, China; b Department of Breast Surgery, The First Hospital of China Medical University, Shenyang, Liaoning 110000, China; c Department of Ultrasound, The First Hospital of China Medical University, Shenyang, Liaoning 110000, China; d Department of Pathology, The First Hospital of China Medical University and College of Basic Medical Sciences, Shenyang, Liaoning 110000, China

Metabolic reprogramming is a key feature of tumor cells and plays a key role in the adaptation of tumor cells to increased demands of biosynthesis and rapid proliferation.^1^ Numerous studies have shown that some key metabolic enzymes are essential for the initiation and progression of breast cancer (BC). These metabolic enzymes are involved in the regulation of many biological processes such as gene transcription, post-translational modification, and antioxidant capacity of cells, which endow tumor cells with the ability to adapt to divergent environmental stimuli.^2^ Therefore, it is of great significance to identify the role of important metabolic enzymes in the occurrence and development of BC to determine promising therapeutic targets. Free fatty acids are absorbed by cells and esterified with CoA to form acyl-CoA, which can be used as substrates for fatty acid oxidation or lipid synthesis. Acyl-CoA thioesterases (ACOTs) catalyze the hydrolysis of acyl-CoA to produce fatty acids and CoA in cells, thus maintaining the ratio of activated fatty acids to free fatty acids and the content of CoA in cells. ACOT7 exhibits broad specificity; it is active towards fatty acyl-CoAs with long chain lengths and has maximal activity toward arachidonic acid-CoA.^3^ It has been demonstrated that ACOT7 was the only member of ACOTs to be significantly up-regulated compared with non-tumoral BC tissues based on the GEPIA database. However, the precise role of ACOT7 in BC occurrence and development is still unknown. We found that the mRNA levels of ACOT7 in BC tissues were significantly higher than that in adjacent non-tumor tissues (Fig. 1A). ACOT7 mRNA expression was associated with more advanced clinicopathological parameters, including lymph node metastasis and tumor size (Fig. 1B, C). Kaplan–Meier plotter analysis indicated that a high level of ACOT7 mRNA was correlated with shorter distant metastasis-free survival and overall survival (Fig. S1A, B). Next, we used immunohistochemistry staining to examine the protein level of ACOT7 in human BC tissues in our cohort. Evaluation of ACOT7 expression levels was according to the staining of cytoplasmic ACOT7, and the score of intensity was also shown (Fig. S1C). Combined with the clinicopathological characteristics, we found that a high protein level of ACOT7 was correlated with advanced tumor size, lymph node metastasis, and Ki-67 index (Table S1). Importantly, survival analysis of our cohort indicated that high expression of ACOT7 protein in BC tissues was associated with reduced disease-free survival (*P* = 0.027) and overall survival (*P* = 0.021) (Fig. 1D, E). Next, we tested the protein level of ACOT7 in different BC cell lines (Fig. S1D). To explore the effects of ACOT7 on the proliferation and invasion of BC cells, we first established MDA-MB-231 and MCF-7 cells stably overexpressing ACOT7 by lentiviral infection (LV). Efficiency was verified by Western blot illustrated in Figure S2A. CCK-8 experiments indicated that ACOT7 overexpression increased cell viability in MDA-MB-231 and MCF-7 cells (Fig. S2B). EdU analysis demonstrated that ACOT7 overexpression enhanced the proliferative capabilities of MDA-MB-231 and MCF-7 cells (Fig. 1F; Fig. S2C). The transwell test demonstrated that ACOT7 overexpression remarkedly increased the invasive abilities of MDA-MB-231 and MCF-7 cells (Fig. 1G; Fig. S2D). To investigate the role of ACOT7 in tumor growth *in vivo*, MDA-MB-231 cells stably transfected with LV-ACOT7 RNA were subcutaneously implanted into BALB/c nude mice. One week later, tumor volumes were measured every seven days. On the 28th day, the mice were euthanized and tumor weights were measured (Fig. 1H). Compared with the control groups, ACOT7 overexpression in MDA-MB-231 cells significantly increased the volume and size of subcutaneous tumors in mice (Fig. 1I, J). Tumor xenografts of the LV-ACOT7 group displayed elevated expression of ACOT7 protein level and a significant increase in the abundance of Ki-67 positive cells (Fig. S2E, F). To further validate the effects of ACOT7 on the biological properties of BC cells, we then down-regulated ACOT7 expression in MDA-MB-231 and MCF-7 cells using RNA interference. Transfection efficiency was measured by Western blot analysis shown in Figure S3A. To investigate the role of ACOT7 in BC cell proliferation, we performed CCK8 and Edu experiments to measure changes in cell proliferation after the down-regulation of ACOT7 expression levels in MDA-MB-231 and MCF-7 cells. CCK-8 experiments indicated that ACOT7 knockdown decreased cell viability in MDA-MB-231 and MCF-7 cells (Fig. S3B). Similarly, EdU experiments demonstrated that ACOT7 knockdown impaired the proliferative ability of in MDA-MB-231 and MCF-7 cells (Figure S3C, D). The transwell test indicated that the invasion abilities of MDA-MB-231 and MCF-7 cells were significantly decreased after ACOT7 knockdown (Figure S3E, F).

**Figure 1:**
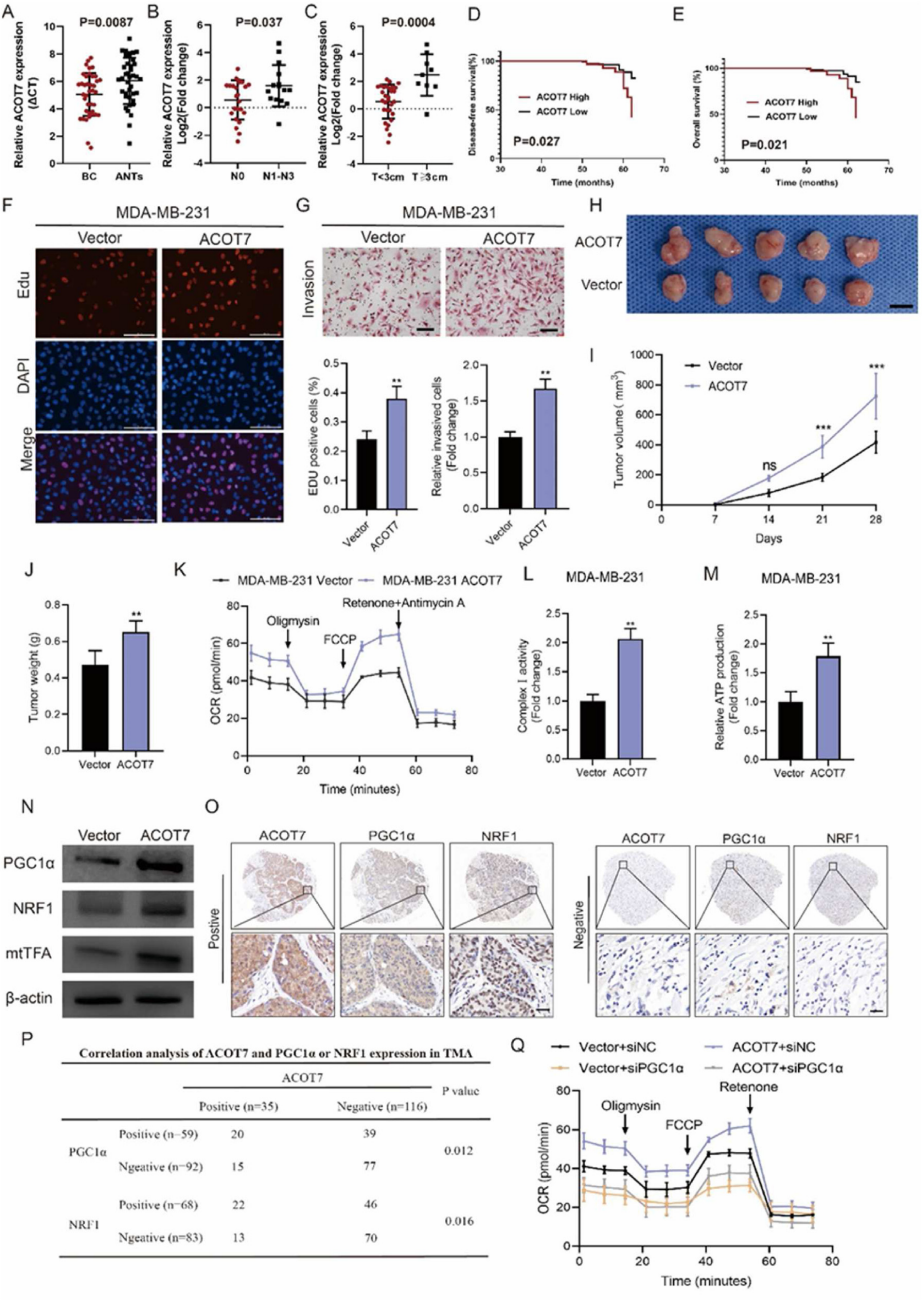
ACOT7 is oncogenic in breast cancer by promoting oxidative phosphorylation via PGC1α. **(A)** Analysis of ACOT7 mRNA level in unpaired breast cancer and normal tissues. A higher Ct value indicated a low mRNA expression. The data were shown as mean ± standard deviation (SD). **(B, C)** Correlation analysis of ACOT7 mRNA level and clinicopathological factors in breast cancer patients. The data were shown as mean ± SD. **(D, E)** The prognostic significance of ACOT7 for breast cancer patients from our tissue microarrays cohort assessed via Kaplan–Meier analysis. Breast cancer patients with high ACOT7 expression had poorer disease-free survival and overall survival than breast cancer patients with low ACOT7 expression. **(F)** Cell proliferation detected by Edu assays (mean ± SD, *n* = 3). ^∗∗^*P* < 0.01. Scale bars, 100 μm. **(G)** Cell invasion tested by transwell assay (mean ± SD, *n* = 3). ^∗∗^*P* < 0.01. Scale bars, 50 μm. **(H)** ACOT7 overexpression or vector MDA-MB-231 cells were subcutaneously injected into female nude mice to observe tumor growth. **(I)** Each group's growth curves of xenograft tumors (mean ± SD, *n* = 5). ^∗∗∗^*P* < 0.001. **(J)** Each group's xenograft tumor weight (mean ± SD, *n* = 5). ^∗∗^*P* < 0.01. **(K)** Oxygen consumption rate measured by Seahorse analysis in control and ACOT7-overexpressing MDA-MB-231 cells (mean ± SD, *n* = 3). **(L)** Analysis of complex I activity in MDA-MB-231 with ACOT7 overexpression (mean ± SD, *n* = 3). ^∗∗^*P* < 0.01. **(M)** Analysis of ATP levels in MDA-MB-231 with ACOT7 overexpression (mean ± SD, *n* = 3). ^∗∗^*P* < 0.01. **(N)** Western blotting was performed to determine PGC1α, NRF1, and mtTFA expression levels in BC cells after ACOT7 overexpression. **(O)** Representative images of immunohistochemical staining of ACOT7, PGC1α, and NRF1 protein expression in human BC tissues were shown. Scale bar, 100 μm. **(P)** Correlation analysis of ACOT7 expression and PGC1α or NRF1 expression in BC tissues in our cohort. **(Q)** PGC1α knockdown abrogated the effects of ACOT7 overexpression on the oxygen consumption rate measured by Seahorse analysis (mean ± SD, *n* = 3).

To understand the molecular basis of the oncogenic properties of ACOT7, we performed GSEA analysis between the ACOT7-high expression group and the ACOT7-low expression group on the transcriptomic data from the TCGA dataset. Indeed, ACOT7-high BCs showed significant enrichment in genes involved in the proteasome, pyrimidine metabolism, pentose phosphate pathway, cell cycle, Parkinson's disease, Huntington's disease, oxidative phosphorylation (OXPHOS), DNA replication, homologous recombination, and glyoxylate and dicarboxylate metabolism (Fig. S4A). OXPHOS provides ATP for cells by transporting electrons to a series of transmembrane protein complexes located at the inner membrane of mitochondria. Up-regulation of glycolysis in tumor cells compared with normal cells has led to the consumption that OXPHOS is commonly impaired in tumor cells. However, multiple studies have shown that OXPHOS is up-regulated in certain tumor types, including BC, leukemia, lymphoma, pancreatic ductal adenocarcinoma, and endometrial cancer.^4^ We first measured the oxygen consumption rate by Seahorse analysis. ACOT7 overexpression significantly increased the oxygen consumption rate in MDA-MB-231 (Fig. 1K). Specifically, ACOT7 overexpression significantly increased both basal and maximal respiratory capacity in MDA-MB-231 cells (Figure S4B, C). Similarly, ACOT7 overexpression significantly increased the oxygen consumption rate in MCF-7 with elevated basal and maximal respiratory capacity (Fig. S4D–F). We next investigated the alterations of ETC complex I activity induced by manipulation of ACOT7 expression. As shown in Figure 1L and S4G, ACOT7 overexpression significantly enhanced the complex Ⅰ activity of MDA-MB-231 and MCF-7 cells. In addition, ACOT7 overexpression remarkedly enhanced ATP production of MDA-MB-231 and MCF-7 cells (Fig. 1M; Fig. S4H). Mitochondria is not only responsible for ATP production but also represents a source of cellular reactive oxygen species (ROS). Previous studies have demonstrated that ROS production is related to mitochondrial membrane potential. Elevation of mitochondrial membrane potential resulting from dysfunctional electron transport enhances ROS generation. We also found a significant elevation in cellular ROS in MDA-MB-231 and MCF-7 cells after ACOT7 knockdown as shown in Figure S5A. Mitosox staining was further used to determine the mitochondrial ROS level after ACOT7 inhibition. We found that ACOT7 inhibition significantly increased the mitochondrial ROS level in MDA-MB-231 and MCF-7 cells (Fig. S5B). We measured mitochondrial membrane potential by JC-1 staining in MDA-MB-231 and MCF-7 cells with ACOT7 knockdown. It has been shown that ACOT7 knockdown significantly decreased JC-1 ratio in MDA-MB-231 and MCF-7 cells (Fig. S5C). Peroxisome proliferator-activated receptor-γ coactivator 1α (PGC1α) is a key transcriptional coactivator that regulates mitochondrial gene expression, and directly determines mitochondrial function, including mitochondrial respiration, biosynthesis, and redox capacity by interacting with nuclear respiratory factors NRF1, NRF2, estrogen associated receptor α, PPARα, and PPARγ. It has been demonstrated that PGC1α binds to and activates NRF1 to mediate co-transcriptional activation of mtTFA, TFB1M, TFB2M, and other genes, thereby regulating the expression of mitochondrial DNA-encoded proteins. Western blot was used to analyze the expression levels of the PGC1α, NRF1, and mtTFA in BC cells after manipulating ACOT7 expression. ACOT7 overexpression augments the expression of PGC1α, NRF1, and mtTFA (Fig. 1N). ACOT7 knockdown decreased the expression of PGC1α, NRF1, and mtTFA (Fig. S6A). We further evaluated the expression correlation between ACOT7, PGC1α, and NRF1 in our TMA cohort by immunohistochemistry analysis (Fig. 1O). ACOT7 expression was found to be positively correlated with PGC1α and NRF1 expression (Fig. 1P). Similarly, immunofluorescence analysis further verified that ACOT7 knockdown reduced the protein level of PGC1α, NRF1, and mtTFA, whereas ACOT7 overexpression increased the expression of PGC1α, NRF1, and mtTFA (Fig. S6B–D).

Next, we used RNA interference targeting PGC1α in ACOT7-overexpressed BC cells. The transfection efficiency was verified by Western blot (Fig. S7A). EdU experiments demonstrated that PGC1α knockdown abolished the proliferative ability of BC cells (Figure S7B, C). In transwell assays, PGC1α knockdown reduced the increased invasive ability of BC cells induced by ACOT7 overexpression (Fig. S7D, E). To further evaluate the effects of PGC1α on ACOT7-mediated mitochondrial alterations, Seahorse analysis verified that increased basal and maximal oxygen consumption rate caused by ACOT7 overexpression was abrogated by PGC1α knockdown (Fig. 1Q; Figure S8A, B). The silence of PGC1α also abolished the effects of ACOT7 overexpression on the increased ETC complex I activity and ATP production (Figure S8C, D). The regulatory effect of ACOT7 on PGC1α is required for further investigations. It has been reported that ACOT7 may play a regulatory role by altering the cellular levels of fatty acid ligands for certain transcription factors. It has been demonstrated that fatty acids function as potential ligands for PPARα activity for functional signaling by the PPARα–PGC1α complex, which, in turn, activates mitochondrial biogenesis and OXPHOS.^5^ Considering that ACOT7 with highest activity towards arachidonic acid-CoA, we tested the expression of PGC1α in ACOT7-silencing cells after arachidonic acid treatment (Fig. S9A). We found that ACOT7 knockdown reduced the protein level of PGC1α, and exogenous arachidonic acid addition (10 μM/L) could rescue the effect of ACOT7 silencing (Fig. S9B). Thus, ACOT7 may regulate PGC1α expression by altering the cellular levels of fatty acids.

ACOT7 inhibition may be a promising anti-tumor strategy for BC cells with high ACOT7 expression. However, ACOT7 inhibition may be ineffective for ACOT7-deficient BC cells. Cytosolic phospholipase A2 (cPLA2) activation accounts for the high levels of arachidonic acids detected in cancer. Once cPLA2 is localized to the membrane, substrates could bind to the active site to produce arachidonic acids. In ACOT7-deficient breast tumors, whether cPLA2α activation may increase arachidonic acid level to activate PGC1α for BC progression is worthy of further exploration. In ACOT7-deficient BC cells, it is also worthy to explore the effects of cPLA2α inhibitors on growth inhibition, which may provide a therapeutic strategy for ACOT7-deficient tumors. Further studies will be conducted to find strategies to target ACOT7-deficient tumors.

Our current study indicated the critical role of ACOT7 in the development of BC by promoting OXPHOS possibly depending on the regulation of PGC1α. These findings warrant further investigations on how ACOT7 affects PGC1α expression to exert its effect on mitochondrial respiration.

## Ethics declaration

All databases we used are publicly available, and our study was performed corresponding to the guidelines of these databases. Tissue samples were approved by the institutional review board of the First Affiliated Hospital of China Medical University.

## Author contributions

XYS conducted experiments, analyzed data, and wrote the manuscript. XYS, QZ, and YYX conceived and designed the experiments. MZW, LTY, XL, and HYC supervised and coordinated the work. All authors reviewed the manuscript and agreed with the results.

## Conflict of interests

All authors declare that there is no potential conflict of interests.

## Funding

This work was supported by the Natural Science Foundation of Liaoning Province of China (No. 2022-YGJC-68), the National Natural Science Foundation of China (No. 81773083), the Scientific and Technological Innovation Leading Talent Project of Liaoning Province of China (No. XLYC1802108), Shanghai Cancer Prevention and Anti-cancer Development Foundation-Hengrui Research Project (China) (No. CYBER-2021-A02). These projects provided all the funds necessary for the collection of cases, the analyses of results, the statistical interpretation of the data, and the submission of the manuscript.
