# Supplementary material for: Acyl-CoA thioesterase 7 is oncogenic in breast cancer by promoting oxidative phosphorylation via PGC1α
Source: Genes Dis. 2023 Oct 20;11(5):101149. doi: 10.1016/j.gendis.2023.101149 (PMC11103411; doi:10.1016/j.gendis.2023.101149)
Supplement: Multimedia component 1 [file mmc1.docx]

**Supplementary Materials**

Page Number

Supplementary Figure 1 2

Supplementary Figure 2 3

Supplementary Figure 3 4

Supplementary Figure 4 5

Supplementary Figure 5 6

Supplementary Figure 6 7

Supplementary Figure 7 8

Supplementary Figure 8 9

Supplementary Figure 8 10

Supplementary Table 1 11

Supplementary Materials and Methods 12-15


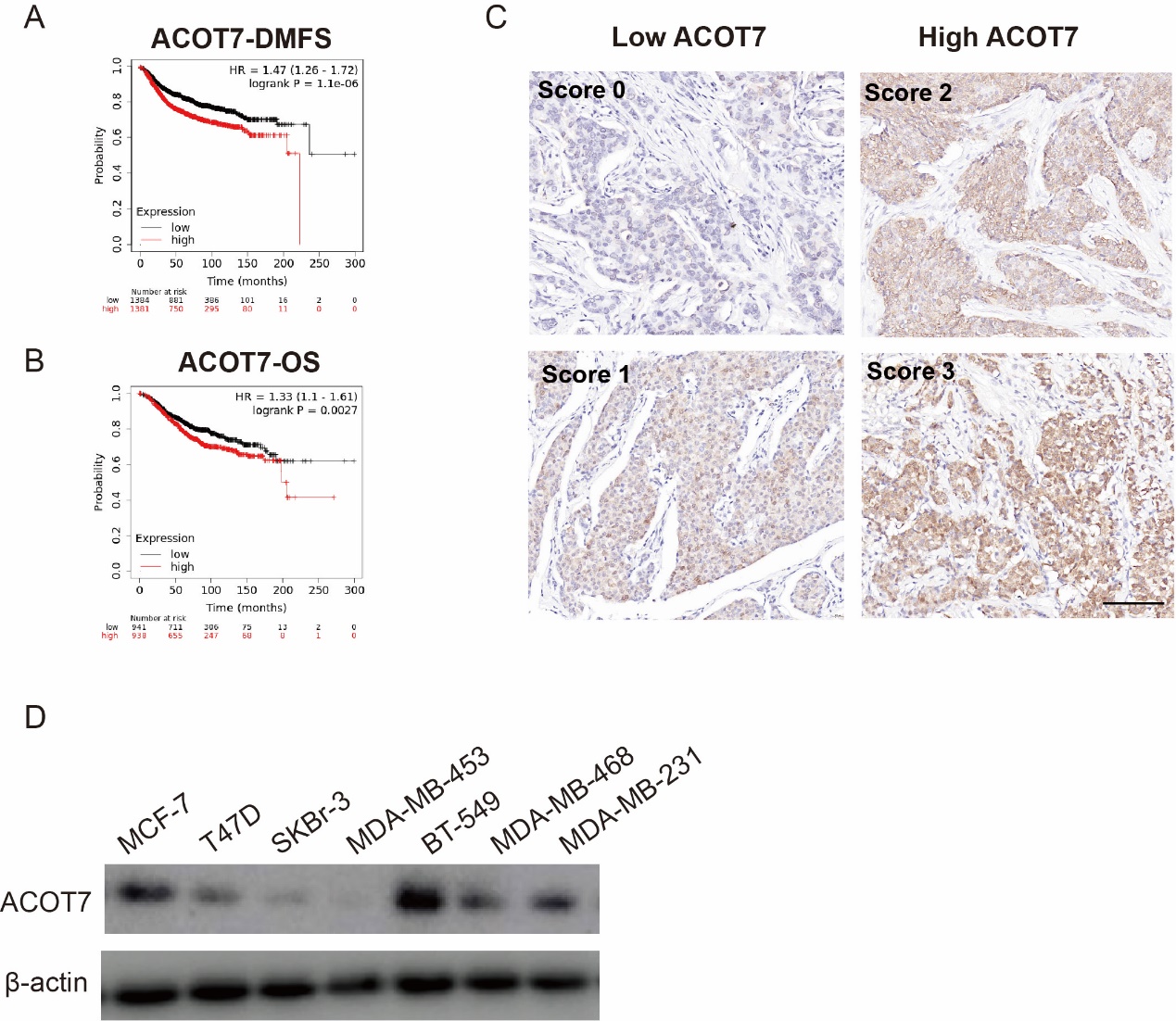
Fig S1: (A) Kaplan-Meier Plotter analysis of ACOT7 mRNA and distant metastasis-free survival in breast cancer patients. (B) Kaplan-Meier Plotter analysis of ACOT7 mRNA and overall survival in breast cancer patients. (C) Representative images of immunohistochemical (IHC) staining of ACOT7 with different score of intensity. Scale bar, 100 µm. (D) The protein level of ACOT7 in different BC cell lines verified by western blot.


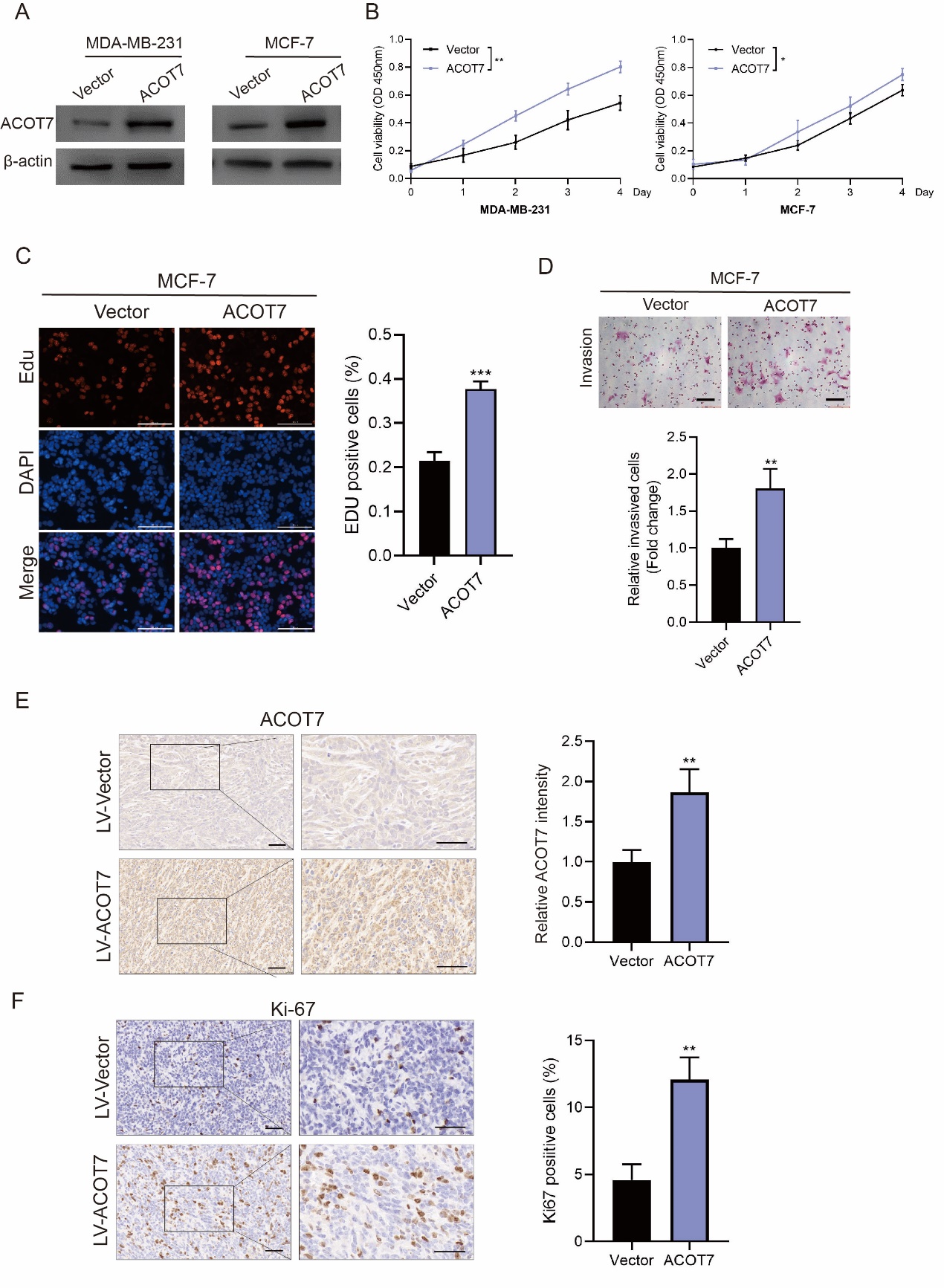
Fig S2: (A) Efficiency of ACOT7 overexpression in MDA-MB-231 and MCF-7 cells verified by western blot. (B) Cell viability detected by CCK-8 assays in MDA-MB-231 and MCF-7 cells (mean ± SD, n = 3). *P < 0.05, **P < 0.01. (C) Cell proliferation detected by Edu assays (mean ± SD, n = 3). ***P < 0.001, Scale bars, 100 µm. (D) Cell invasion tested by Transwell assay (mean ± SD, n = 3). **P < 0.01. Scale bars, 50 µm. (E) Immunohistochemical staining of ACOT7 in tumor sections from each group (mean ± SD, n = 5). **P < 0.01. (F) Immunohistochemical staining of Ki-67 in tumor sections from each group (mean ± SD, n = 5). **P < 0.01.


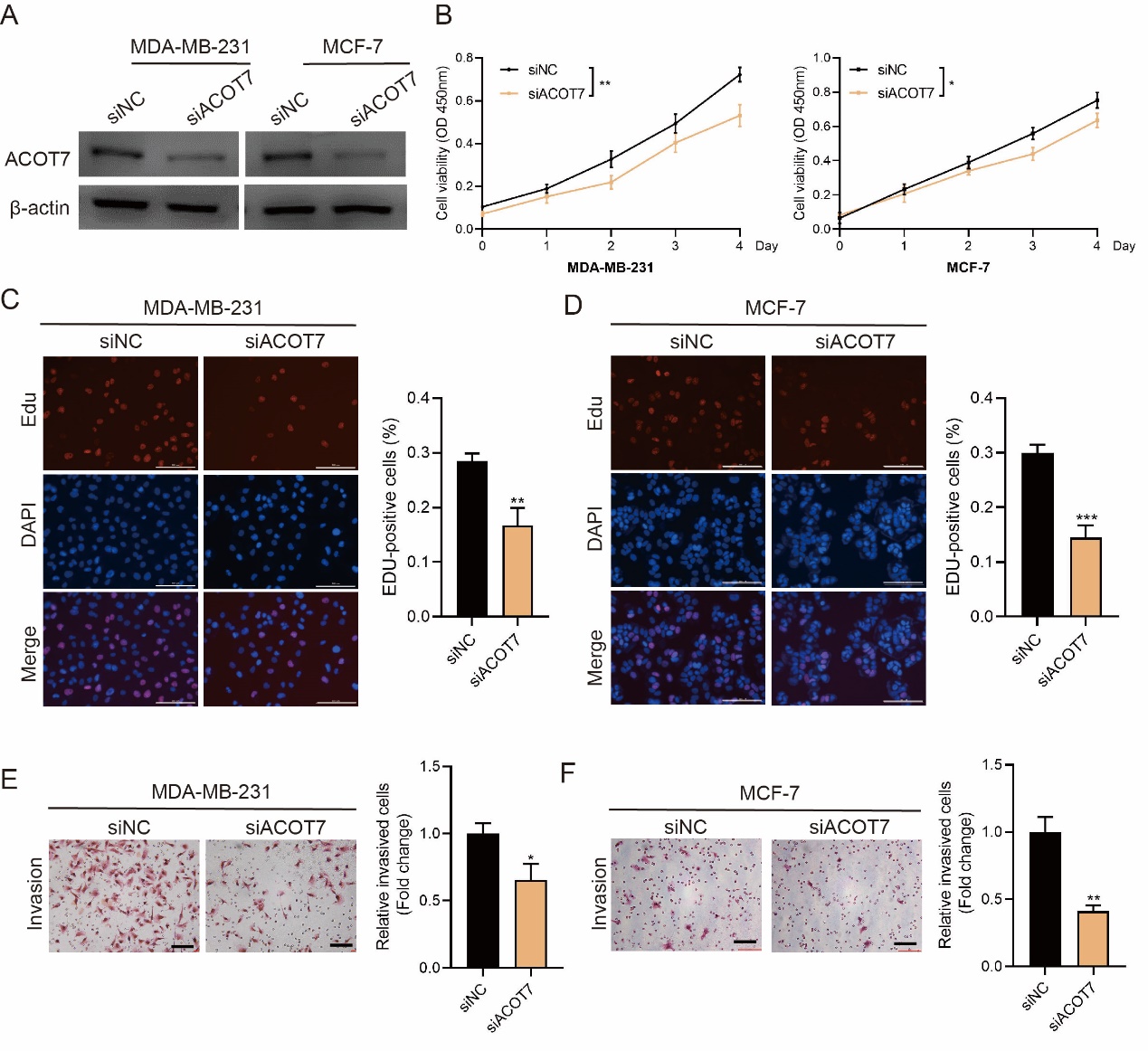


Fig S3: (A) Efficiency of ACOT7 knockdown in MDA-MB-231 and MCF-7 cells verified by western blot. (B) Cell viability detected by CCK-8 assays in MDA-MB-231 and MCF-7 cells (mean ± SD, n = 3). *P < 0.05, **P < 0.01. (C-D) Cell proliferation detected by Edu assays in MDA-MB-231 and MCF-7 cells (mean ± SD, n = 3). **P < 0.01, ***P < 0.001, Scale bars, 100 µm. (E-F) Cell invasion tested by Transwell assay (mean ± SD, n = 3). *P < 0.05, **P < 0.01. Scale bars, 50 µm.


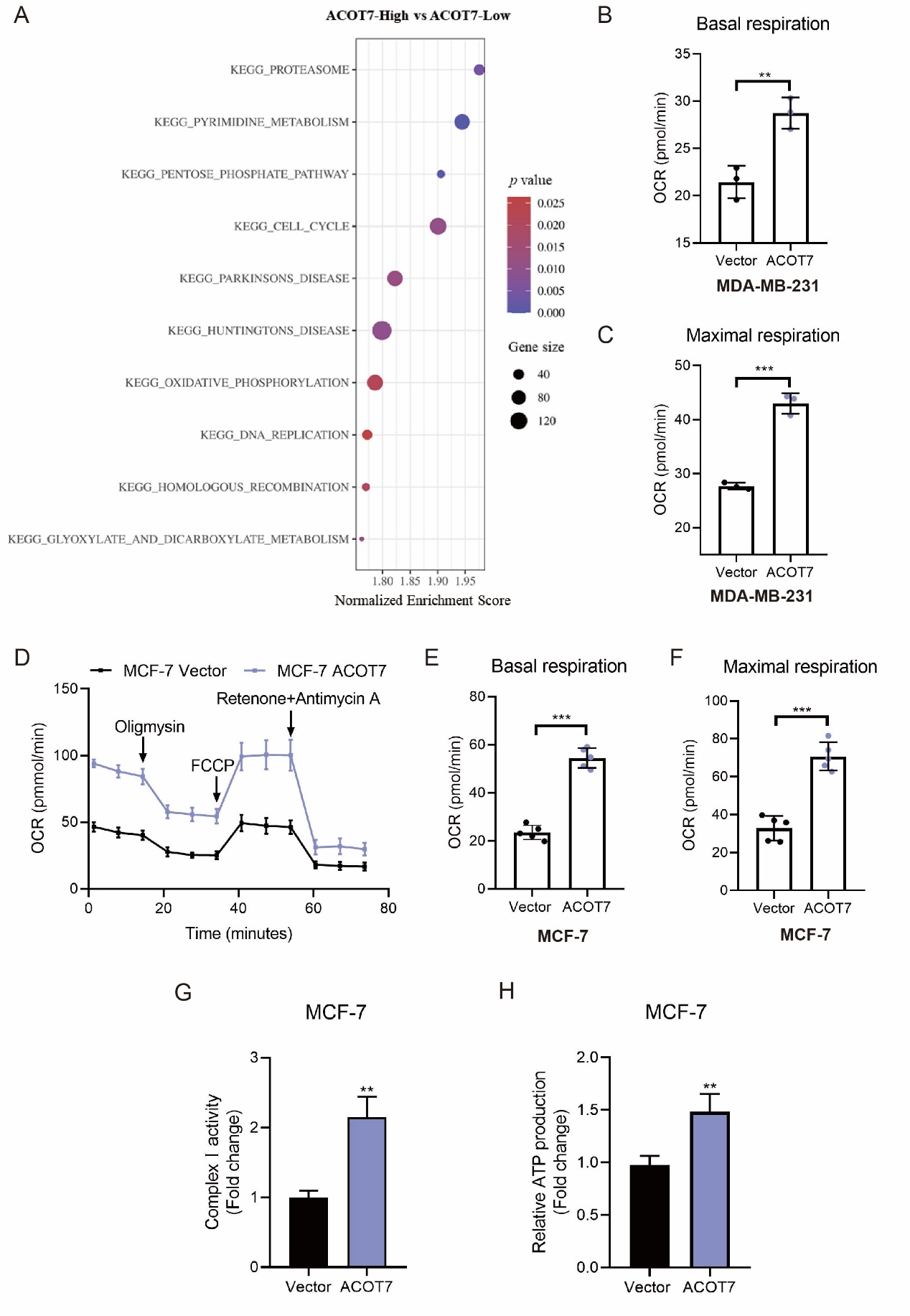
Fig S4: (A) Normalized enrichment scores of significantly up-regulated pathways identified by gene set enrichment analysis in ACOT7-high and ACOT7-low patients based on the TCGA database. (B-C) Basal respiration rates and maximal respiration rates in control and ACOT7-overexpressing MDA-MB-231 cells (mean ± SD, n = 3). **P < 0.01, ***P < 0.001. (D) Oxygen consumption rates measured by Seahorse analysis in control and ACOT7-overexpressing MCF-7 cells (mean ± SD, n = 3). (E-F) Basal respiration rates and maximal respiration rates in control and ACOT7-overexpressing MCF-7 cells (mean ± SD, n = 3). ***P < 0.001. (G) Analysis of Complex I activity in MCF-7 cells with ACOT7 overexpression (mean ± SD, n = 3). **P < 0.01. (H) Analysis of ATP levels in MCF-7 cells with ACOT7 overexpression (mean ± SD, n = 3). **P < 0.01.


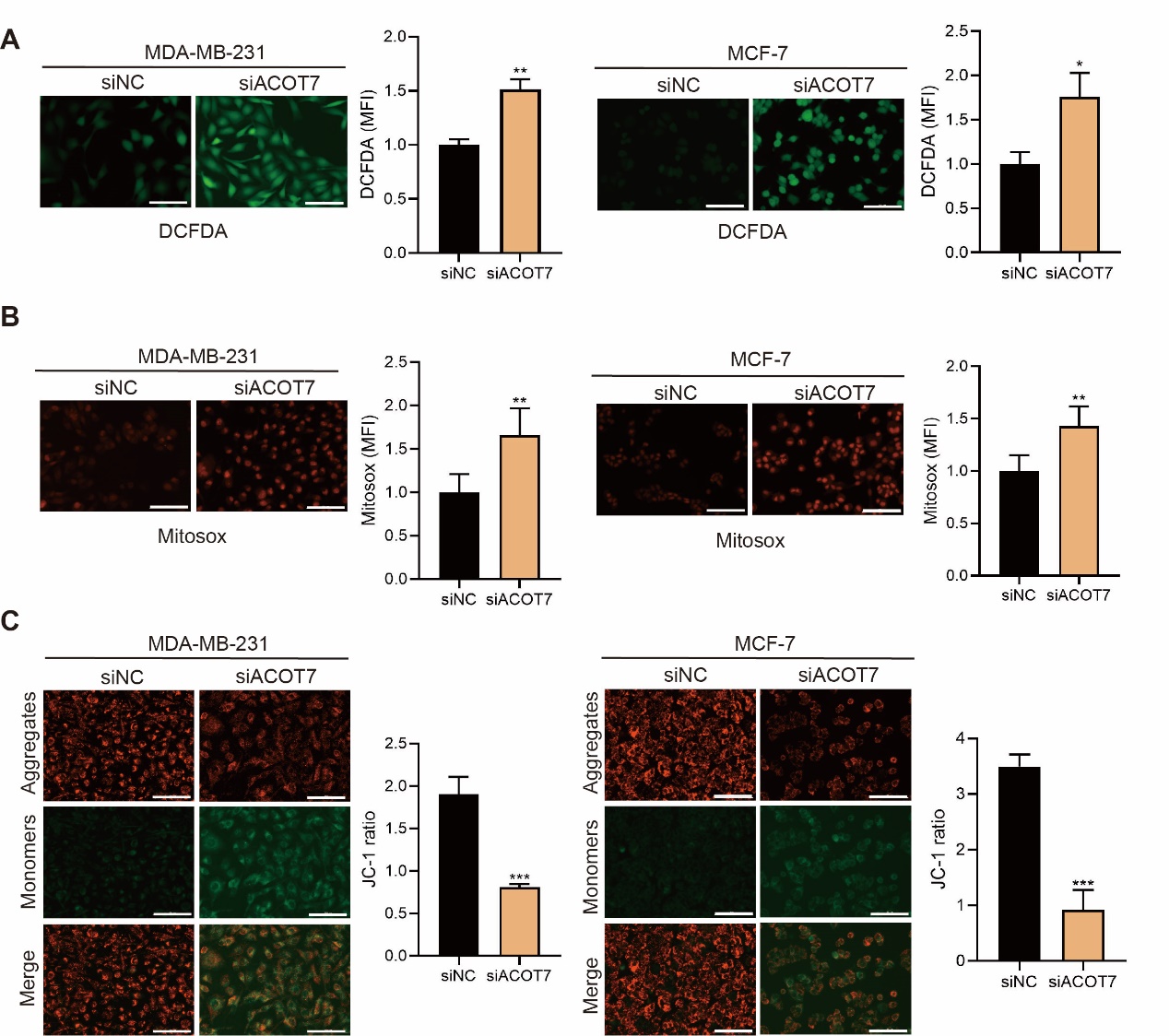


Fig S5: (A) Cellular ROS intensity measured by DCFH-DA fluorescence probe in MDA-MB-231 and MCF-7 cells with ACOT7 knockdown (mean ± SD, n = 3). *P < 0.05, **P < 0.01. Scale bars, 100 µm. (B) Mitochondrial ROS intensity measured by MitoSOX fluorescence probe in MDA-MB-231 and MCF-7 cells with ACOT7 knockdown (mean ± SD, n = 3). **P < 0.01. Scale bars, 100 µm. (C) Measurements of mitochondrial membrane potential by JC-1 staining in MDA-MB-231 and MCF-7 cells with ACOT7 knockdown (mean ± SD, n = 3). ***P <0.001. Scale bars, 100 µm.


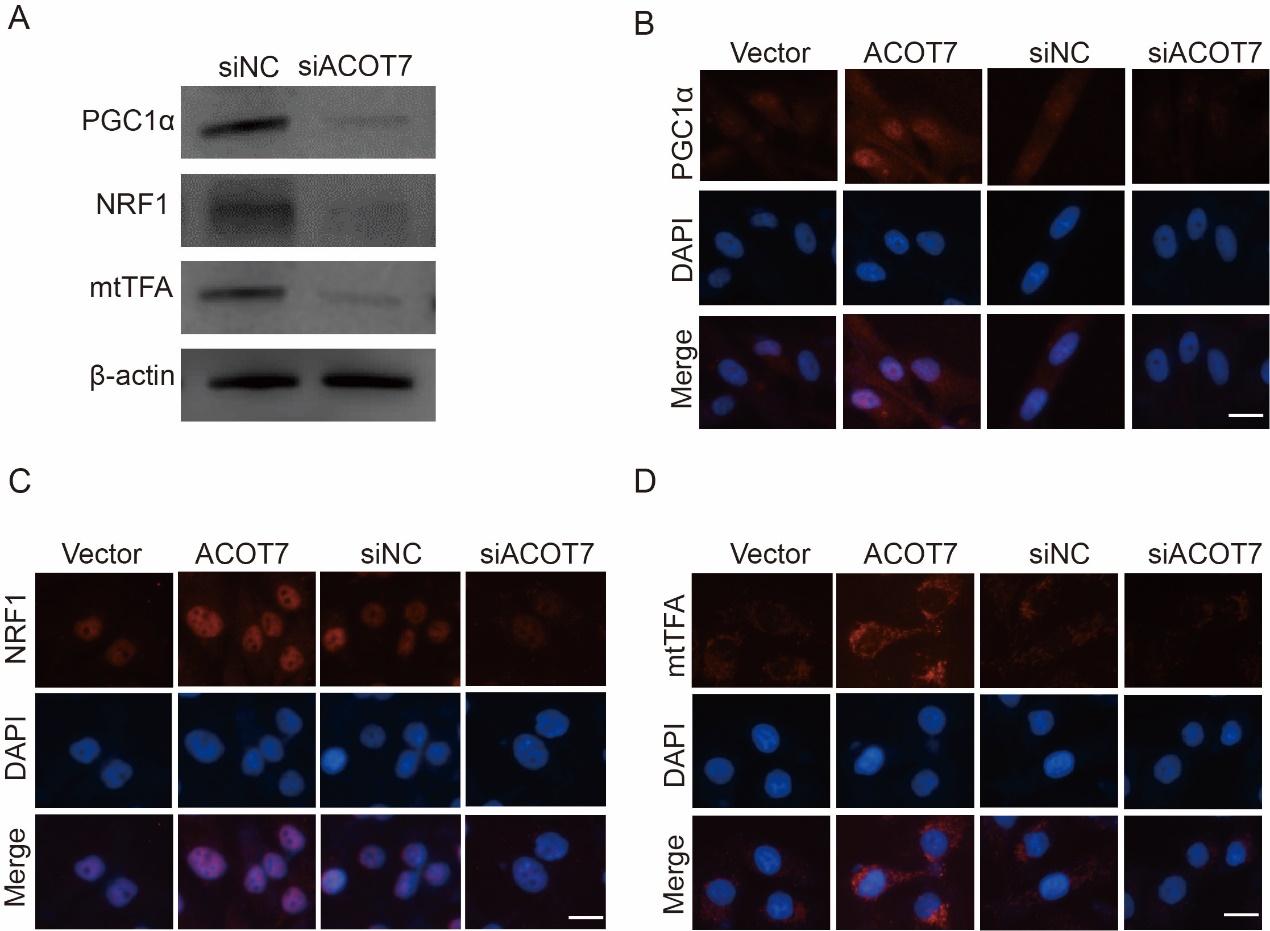


Fig S6: (A) PGC1α, NRF1 and mtTFA expression level in MDA-MB-231 cells after ACOT7 knockdown verified by western blot. (B-D) Immunofluorescence assays were performed to analyze PGC1α (B), NRF1 (C) and mtTFA (D) expression level in MDA-MB-231 cells after ACOT7 overexpression or knockdown. Scale bars, 20 µm.


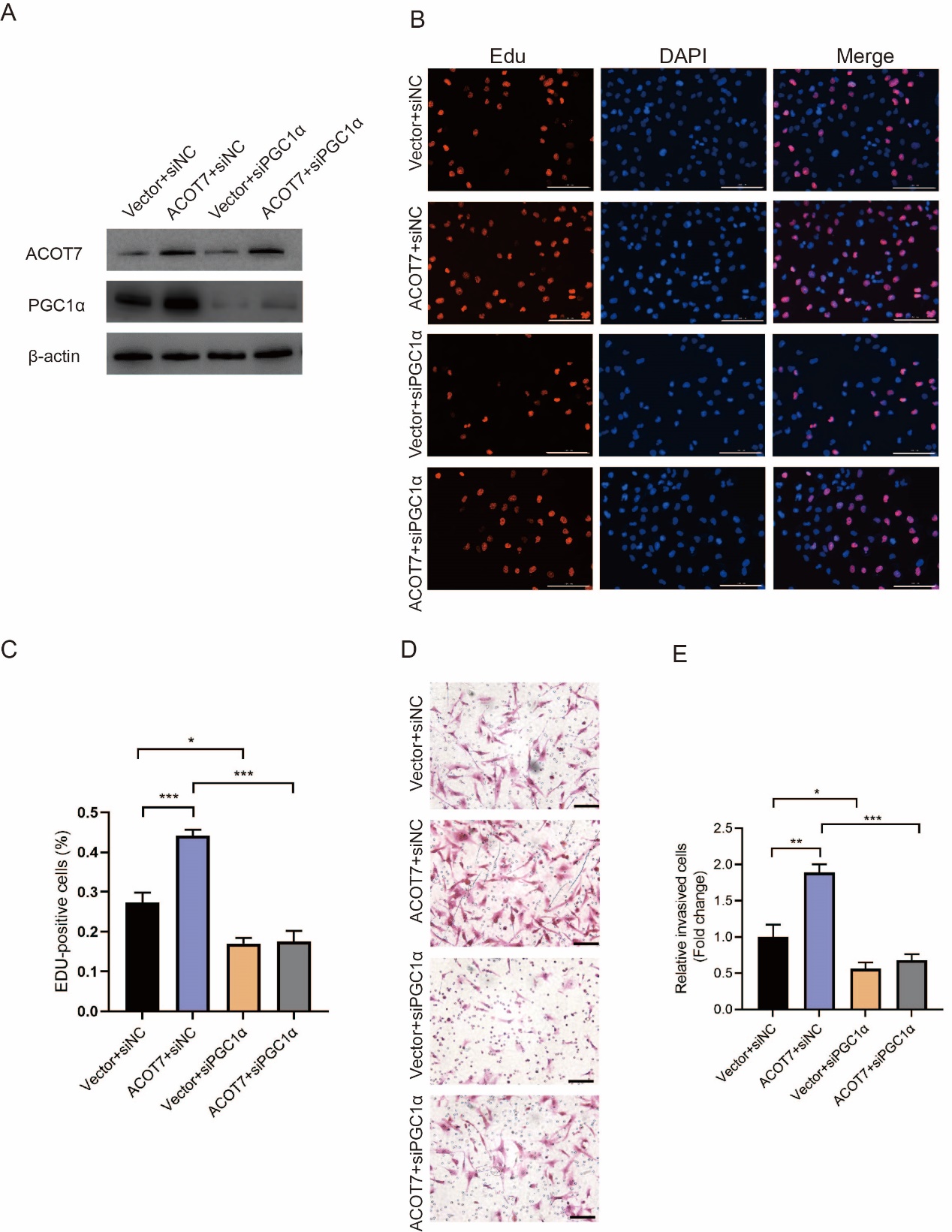


Fig S7: (A) Efficiency of PGC1α knockdown verified by western blot. (B-C) PGC1α knockdown impaired the promotion of BC cell proliferation induced by ACOT7 overexpression (mean ± SD, n = 3). Scale bars, 100 µm. *P < 0.05, *** P <0.001. (D) PGC1α knockdown impaired the promotion of BC cell invasion induced by ACOT7 overexpression (mean ± SD, n = 3). Scale bars, 50 µm. *P < 0.05, **P < 0.01, *** P <0.001.


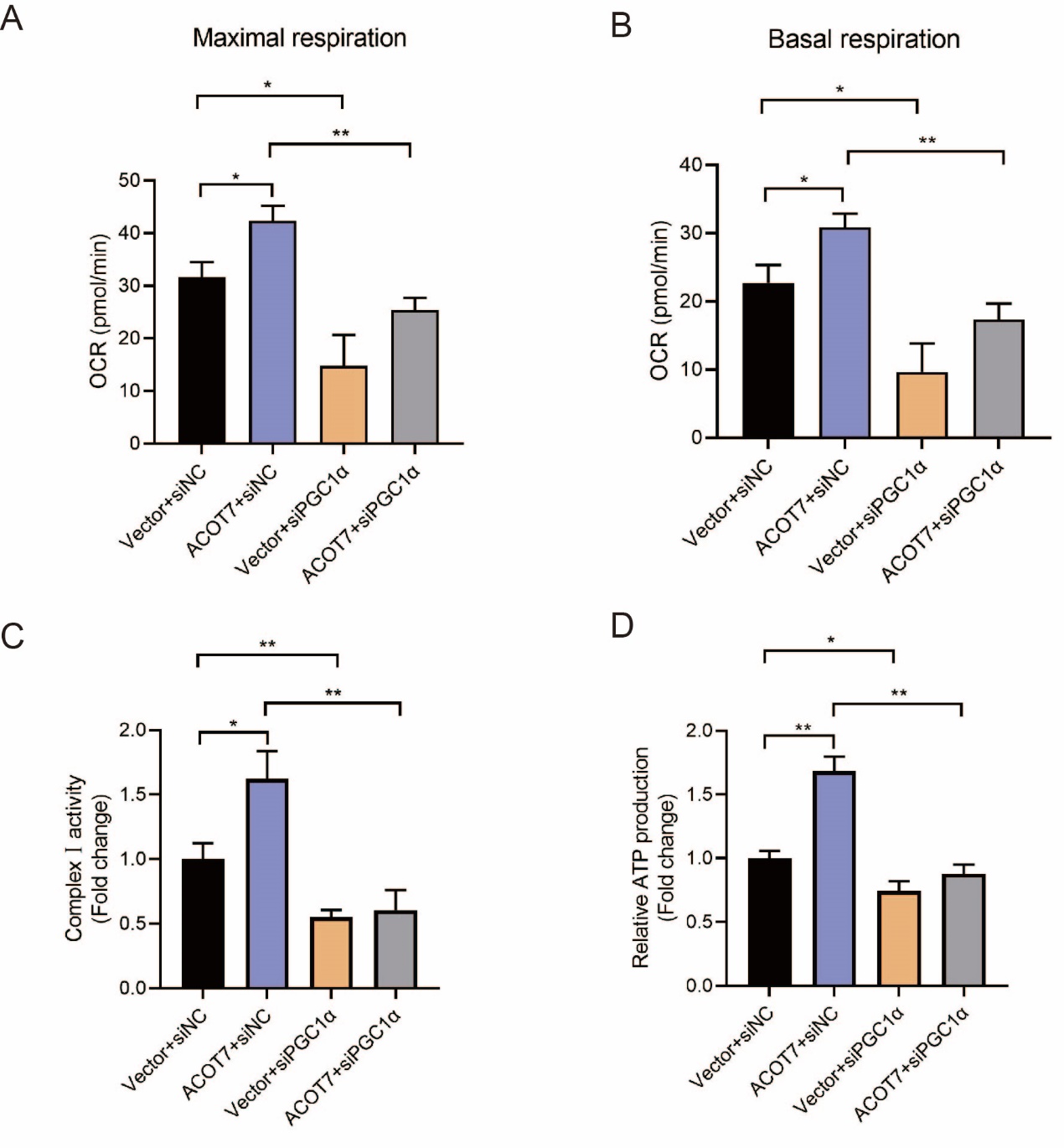
 Fig S8: (A) PGC1α knockdown abrogated the effects of ACOT7 overexpression on the maximal respiratory capacity (mean ± SD, n = 3). *P < 0.05, **P < 0.01. (B) PGC1α knockdown abrogated the effects of ACOT7 overexpression on the basal respiratory capacity (mean ± SD, n = 3). *P < 0.05, **P < 0.01. (C) PGC1α knockdown abrogated the effects of ACOT7 overexpression on the ETC complex I activity (mean ± SD, n = 3). *P < 0.05, **P < 0.01. (D) PGC1α knockdown abrogated the effects of ACOT7 overexpression on the ATP production (mean ± SD, n = 3). *P < 0.05, **P < 0.01.


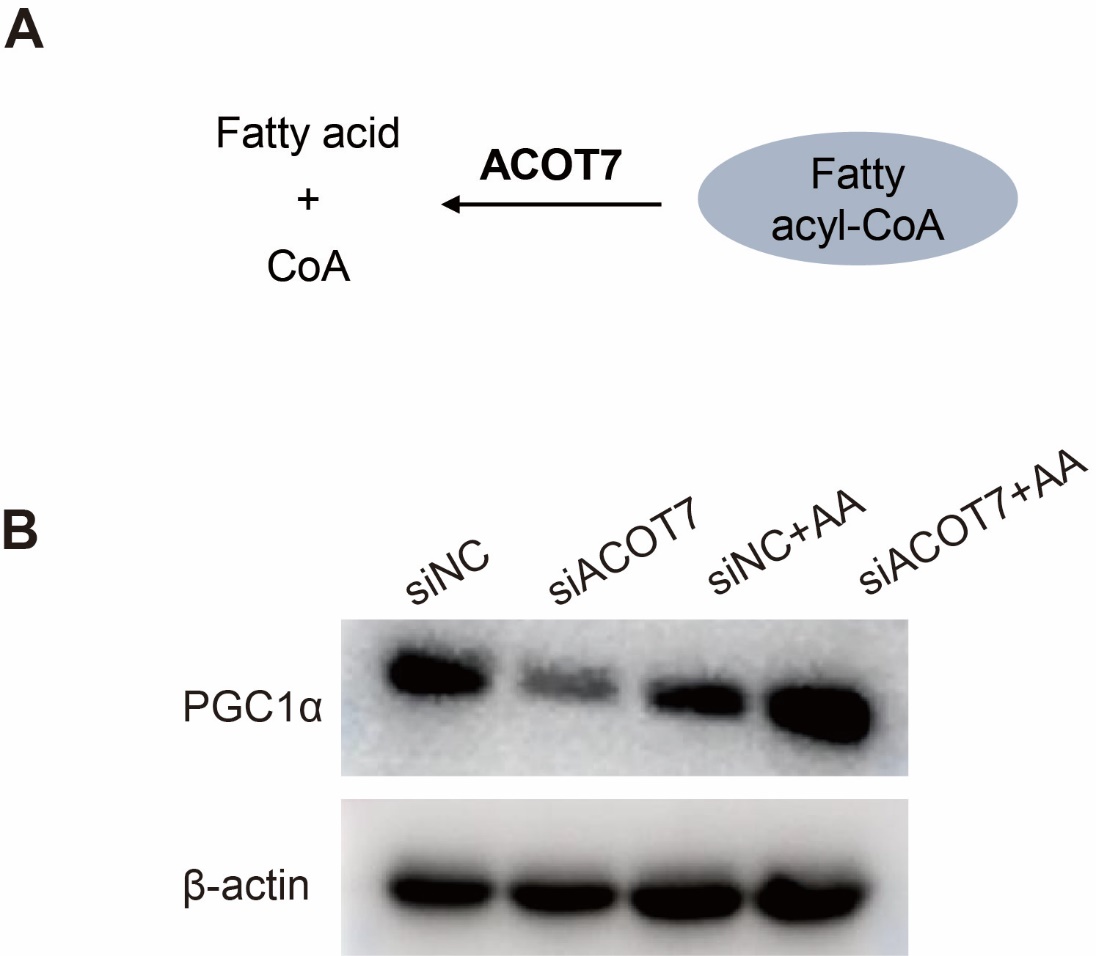


Fig S9: (A) Diagram of metabolism of ACOT7 in cells. (B) The expression of PGC1α in ACOT7-silencing cells after AA treatment verified by western blot.

Table S1: Clinicopathological characteristics of breast cancer patients in the tissue microarrays.

| Variables | | Cases(n=151) | | ACOT7  Low | ACOT7  High | P value |
| --- | --- | --- | --- | --- | --- | --- |
| Tumor size |  | |  | |  | **0.032*** |
| ≤3cm | 80 | | 67 | | 13 |  |
| >3cm | 71 | | 49 | | 22 |  |
| Lymph node metastasis |  | |  | |  | **0.022*** |
| N0 | 50 | | 44 | | 6 |  |
| N1-3 | 101 | | 72 | | 29 |  |
| Molecular subtype |  | |  | |  | 0.108 |
| Luminal A | 43 | | 38 | | 5 |  |
| Luminal B | 95 | | 67 | | 28 |  |
| HER2+ | 4 | | 3 | | 1 |  |
| TNBC | 9 | | 8 | | 1 |  |
| ER |  | |  | |  | 0.836 |
| Positive | 131 | | 101 | | 30 |  |
| Negative | 20 | | 15 | | 5 |  |
| PR |  | |  | |  | 0.929 |
| Positive | 120 | | 92 | | 28 |  |
| Negative | 31 | | 24 | | 7 |  |
| HER2 |  | |  | |  | 0.810 |
| Positive | 45 | | 34 | | 11 |  |
| Negative | 106 | | 82 | | 24 |  |
| Ki-67 |  | |  | |  | **0.027*** |
| >14% | 107 | | 77 | | 30 |  |
| ≤14% | 44 | | 39 | | 5 |  |

**Supplementary Materials and Methods**

**Cell culture and treatment**

Human BC cell lines MCF-7, T47D, SKBr-3, MDA-MB-453, BT549, MDA-MB-468, and MDA-MB-231 were obtained from ATCC (Manassas, USA). T47D, MDA-MB-468 and MDA-MB-231 cells were maintained in high-glucose Dulbecco’s modified Eagle’s medium (DMEM; HyClone) with 10% foetal bovine serum. MCF-7, SKBr-3, MDA-MB-453 and BT549 cells were maintained in RPMI 1640 medium (Invitrogen, Carlsbad, USA) with 10% fetal bovine serum (FBS; Gibco). Cells were incubated at 37°C with 20% O_2_ and 5% CO_2_ (Thermo, Waltham, USA). Arachidonic acid (AA) was purchased from Sigma.

**RNA isolation and quantitative real-time PCR (RT-qPCR)**

Total RNA from tissues was extracted using TRIzol (Invitrogen, USA). Takara First Strand cDNA Synthesis Kit was used for cDNA synthesis. Relative mRNA levels were normalized to the level of GAPDH and calculated by the 2^-ΔΔCt^ method. ACOT7 forward primer: 5’-CTGCACCCTGCACGGCTTTG-3’, ACOT7 reverse primer: 5’-CGGAAGCTGTGACGATGTTG-3’; GAPDH forward primer: 5’-CGGATTTGGTCGTATTGGG-3’, GAPDH reverse primer: 5’-CTGGAAGATGGTGATGGGATT-3’.

**Immunohistochemistry (IHC)**

A total of 151 primary BC samples were collected in the First Hospital of China Medical University. This study was approved by the Ethics Committee of China Medical University. These patients were diagnosed by two independent pathologists with complete follow-up data. The differences were settled by consensus. Patients with other malignancies were excluded. Firstly, BC samples were dewaxed and hydrated. 3% H_2_O_2_ was used to inactivate endogenous peroxidase. After antigen extraction, sections were incubated with primary antibody ACOT7 (1/100, ab85151, Abcam), PGC1α (1/100, ab191838, Abcam) and NRF1 (1/200, ab175932, Abcam), respectively. The sections were incubated with the secondary antibodies and DAB regents for staining (Zhong Shan Golden Bridge Biotechnology, Beijing, China). The immunoreactivity of ACOT7, PGC1α and NRF1 was scored according to the intensity of staining (negative=0, weak=1, moderate=2, strong=3) and percentage of immunoreactive tumor cells (<5% = 0, 5–25% =1, 25–50%=2, 50–75% = 3, >75%=4). The final score was calculated by multiplying the above two scores (ranging from 0 to 12). Final scores higher than 6 were defined as high expression, while scores equal to or less than 6 were defined as low expression.

**Western blotting (WB)**

Total protein contents were extracted using a Total Protein Extraction Kit (KeyGen Biotech, Nanjing, China). Proteins were separated by a 10% SDS–polyacrylamide gel electrophoresis and transferred onto polyvinylidene difluoride membranes. The membranes were blocked with 5% nonfat milk in TBST and incubated with anti-ACOT7 (1/1000, ab85151, Abcam), anti-PGC1α (1/1000, Abcam, ab191838), anti-NRF1 (1/1000, ab175932, Abcam). anti-mtTFA (1:1000, ab176558, Abcam) or anti-β-actin (1:1000, ab8226, Abcam), respectively. After three washes with TBST, the membranes were incubated with secondary antibodies. Finally, the membranes were imaged by GelCapture software (DNR Bio-Imaging Systems, Jerusalem, Israel).

**Immunofluorescence (IF) staining assay**

Cells grown in 24-well plates were fixed in 4% paraformaldehyde and permeabilized with 0.1% Triton X-100. After three 5min washes with PBS, the samples were then blocked for 1h with 2% bovine serum albumin followed by incubation with PGC1α antibody (1:500, ab191838, Abcam) or NRF1 antibody (1:500, ab175932, Abcam) or mtTFA antibody (1:500, ab176558, Abcam) or anti-γ-H2AX (1:500, 10856-1-AP, Proteintech) overnight at 4°C and then with Dylight594-conjugated secondary antibodies (Abbkine, Wuhan, China) for 1h at room temperature. After co-staining with DAPI, the samples were imaged with a fluorescence microscope.

**Establishment of stable ACOT7-overexpressed BC cell lines**

HEK293T cells were transiently transfected with ACOT7 (LV-ACOT7) and the control group (LV-Vector) (GenePharma, Shanghai, China). After 48h transfection, the supernatants containing lentivirus were collected and mixed with 5μg/ml polybrene to infect MDA-MB-231 and MCF-7cells to improve the infection efficiency. Stable cell lines were selected with 2μg/ml puromycin (Solarbio, Beijing, China). The efficiency of ACOT7 overexpression in BC cells was confirmed by WB.

**RNAi interference**

MDA-MB-231 and MCF-7 cells were transfected with small interfering RNAs targeting ACOT7, PGC1α or their negative control (siNC) (GenePharma, Shanghai, China) using Lipofectamine 3000 reagent (Invitrogen) according to the instructions. Follow-up experiments were performed 48 hours after transfection.

**Cell viability and proliferation assays**

Cell counting kit-8 (CCK-8) assay was used to detect cell viability (Dojindo, Japan). Transfected cells were seeded in 96-well plates at a density of 2×10^3^. Cells in each well were incubated with 10μl of CCK-8 reagent for 1h at 37°C, and the absorbance at 450nm was measured by a microplate reader (Bio-Rad, Hercules, CA, USA) every day for 5 days. To measure cell proliferation, EdU-positive cells were identified by an EdU Cell Proliferation Kit (Beyotime, China) according to the manufacturer’s instructions.

**Cell invasion assays**

Cell invasion assays were carried out using Corning Transwell chambers. The upper chamber was filled with 3×10^4^ cells of each group suspended in 200μl serum-free medium, while the lower chamber contains 20% FBS. After incubation for 24h, the chambers were fixed in 4% paraformaldehyde for 3min and stained with hematoxylin and eosin (H&E). The quantity of stained cells was counted under a microscope (Leica, Germany). For cell invasion assays, Transwell chambers were coated with Matrigel (BD Biosciences, USA).

**Oxygen consumption rate (OCR) measurements**

The OCR was measured using an XF-96 Analyzer (Seahorse Bioscience). For the OCR measurement, stable cell lines were treated with 1µM oligomycin, 0.5µM carbonyl cyanide-p-trifluoromethoxyphenylhydrazone (FCCP), 0.5µM rotenone and 0.5µM antimycin A (XF cell mito stress test kit, Seahorse Bioscience). The transfected cells of each group were seeded into Seahorse XF96 plates at 1×10^4^ per well and incubated at 37°C with 5% CO_2_. The calibration solution was added to the Utility Plate in advance and placed in a CO_2_-free incubator at 37°C overnight. Seahorse XF Base Medium was used to prepare the test solution, and the required substrate was added and incubated at 37°C for later use. After 24 hours, seeding medium was replaced with test solution to incubate transfected cells for 1 h at 37 °C, making transfected cells to pre-equilibrate with the XF base medium. Oligomycin, FCCP, rotenone and antimycin A were prepared and diluted to the suitable concentration according to the instructions. The Seahorse detector first measured the basal OCR value under normal conditions, then added oligomycin, FCCP and rotenone/antamycin A, according to the time points of the instructions. The OCR value was recorded at each time point, and the data was analyzed by Seahorse XF-96 Wave software.

**Detection of reactive oxygen species (ROS)**

Fluorescence probe DCFH-DA (Beyotime, Beijing, China) was used to measure cellular ROS. MitoSOX Red probe (Thermofisher, USA) was used to measure mitochondrial ROS. MDA-MB-231 and MCF-7 BC cells were incubated with DCFH-DA at 37°C for 20 min or MitoSOX Red probe at 37°C for 10 min and then washed by PBS for three times. The images were taken by a fluorescence microscope and the mean intensity was calculated.

**Detection of mitochondrial depolarization**

JC-1 is an ideal fluorescent probe widely used for mitochondrial membrane potential (MMP) detection. At high MMP, JC-1 aggregates in the mitochondrial matrix, forming a polymer (J-Aggregates) and red fluorescence. When the mitochondrial membrane potential is low, JC-1 cannot accumulate in the matrix of mitochondria. forming a monomer and green fluorescence. MMP was tested by a JC-1 assay kit (Beyotime, Beijing, China). MDA-MB-231 and MCF-7 cells were incubated with JC-1 at 37°C for 20 min under the dark. Cells were washed three times and imaged by fluorescence microscopy. The ratios of red to green fluorescence intensity were calculated as MMP.

**Xenograft assays**

Female BALB/C nude mice (4 weeks old) were purchased from Charles River, Beijing. BALB/c mice were randomly assigned into 2 groups (5 mice per group). The stably-transfected MDA-MB-231 cells (5×10^6^ cells per mouse) were re-suspended in 100μl PBS and 100μl matrigel substrate, and subcutaneously injected into the 4th coupled mammary fat pad of mice. Tumor volumes were recorded every seven days. After 28 days, mice were sacrificed. Tumors were harvested and weighed. After the xenografts were imaged, the tumors were fixed in 4% paraformaldehyde and embedded in paraffin. Serial 5μm sections were stained with haematoxylin and eosin (H&E) for validation. The animal studies were approved by the China Medical University Institutional Ethics Committee.

**Gene Set Enrichment Analysis (GSEA) analysis**

GSEA analysis is a computational method used to determine whether a previously defined set of genes exhibits statistically significant differences between two biological states. The results of this paper used two phenotypes divided by ACOT7 mRNA expression levels in the Cancer Genome Atlas (TCGA) dataset (https://portal.gdc.cancer.gov/). The TCGA samples were classified as ACOT7-High and ACOT7-Low group and analyzed with GSEA 2.0.9 software (http://www.broadinstitute.org/gsea/).

**Statistical analyses**

Statistical analyses were performed using GraphPad Prism software (v8.0). Student’s t-test was used to compare two groups, while one way analysis of variance (ANOVA) was used for multiple data groups. For in vitro and in vivo studies, statistical significance was determined by the two-tailed unpaired Student’s t-test. All experiments were repeated at least three times. Bar plots with error bars were presented as mean ± standard deviation (SD). Chi-Square test and Pearson’s χ2 test were used to analyze patient data. A P value < 0.05 was considered as statistically significant difference. Survival analysis was performed using the Kaplan-Meier method and log-rank test.
